# ASO Author Reflections: Enhancing Surgical Decision-Making for Breast Reconstruction—Machine Learning-Driven Prediction of Postoperative Quality of Life

**DOI:** 10.1245/s10434-023-14008-y

**Published:** 2023-07-30

**Authors:** Cai Xu, André Pfob, Chris Sidey-Gibbons

**Affiliations:** 1 Section of Patient Centered Analytics, Division of Internal Medicine, Department of Symptom Research, The University of Texas MD Anderson Cancer Center, Houston, TX USA; 2 MD Anderson Center for INSPiRED Cancer Care (Integrated Systems for Patient-Reported Data), The University of Texas MD Anderson Cancer Center, Houston, USA; 3 Department of Obstetrics and Gynecology, Heidelberg University Hospital, Heidelberg, Germany

## Past

For women undergoing cancer-related mastectomy and reconstruction, treatment decisions play a crucial role in postoperative outcomes. However, the current decision-making process often relies on non-randomized group-level evidence or physician preferences,^1^ potentially resulting in sub-optimal treatment recommendations. Machine learning (ML), an AI technology, has significantly enhanced clinician performance by improving risk predictions and streamlining standardized processes.^2,3^ While most research has focused on image recognition for clinical diagnosis, there is a growing demand for algorithmic support in treatment decision-making. To address this urgent need, this study aims to develop personalized, data-driven decision aids for women undergoing breast reconstruction, with the goal of improving patient-centered care.

## Present

We trained three distinct ML models to predict meaningful changes in health-related quality of life for women undergoing mastectomy and breast reconstruction. This study involved 1454 to 1538 patients from 11 North American study sites, with a 2-year follow-up. To create unbiased clinical algorithms that consider treatment preferences and body image, we excluded socioeconomic and ethnic variables while incorporating baseline patient-reported outcomes (PROs) into our models. The models achieved a good accuracy, with an AUC of up to 0.82. Our findings demonstrate that baseline PROs have a stronger influence on postoperative satisfaction with breasts compared with treatment decisions.^4^ We highlight the potential of ML algorithms in accurately predicting PROs, offering valuable insights for clinical decision-making.

## Future

The integration of ML and PROs enables individualized outcome predictions and shared decision-making. ML algorithms empower clinicians to tailor treatment recommendations confidently. A significant shift toward AI-guided clinical decision-making is expected in the near future. However, challenges remain for ML to fulfill its potential in optimizing clinical care. First, clinical implementation is necessary to validate algorithm effectiveness. Second, addressing biases in training data is crucial for improving algorithm fairness.^5^ We hope that our research initiates the development of truly individualized and data-driven tools to support patient-centered, clinical decision making. The powerful combination of ML and PROs has the potential to change our current way of clinical decision-making.
